# Multiple screw fixation versus cementless bipolar hemiarthroplasty for femur neck fracture using a nationwide hip fracture registry

**DOI:** 10.1038/s41598-021-01046-3

**Published:** 2021-11-02

**Authors:** Jin-Woo Kim, Kyung-Soon Park, Young-Kyun Lee, Ji Wan Kim, Yong-Chan Ha, Seung-Hoon Baek

**Affiliations:** 1 Department of Orthopaedic Surgery, Nowon Eulji Medical Center, Eulji University, Seoul, Korea; 2 Department of Orthopedic Surgery, Center for Joint Disease, Chonnam National University Hwasun Hospital, Hwasun, Korea; 3 Department of Orthopaedic Surgery, Seoul National University Bundang Hospital, Seongnam, Korea; 4 Department of Orthopedic Surgery, Asan Medical Center, Ulsan University, College of Medicine, Seoul, Korea; 5 Department of Orthopaedic Surgery, Chung-Ang University Hospital, Chung-Ang University School of Medicine, Seoul, Korea; 6 Department of Orthopedic Surgery, School of Medicine, Kyungpook National University, Kyungpook National University Hospital, 130, Dongdeok-ro, Jung-Gu, Daegu, 41944 Korea

**Keywords:** Bone, Trauma, Surgery

## Abstract

Cementless bipolar hemiarthroplasty (BHA) recently gained popularity as a treatment for femur neck fracture (FNF), but there have been few studies comparing this with multiple screw fixation (MSF) in the elderly population. The purpose of this study is to compare (1) surgery-related parameters, (2) reoperation rate as a local complication, (3) in-hospital systemic complication rate, and (4) mortality rate at 1 year after MSF and cementless BHA in patients with FNF using nationwide data. Six-hundred sixty-six hips (aged ≥ 50 years) extracted from nationwide Hip Fracture Registry were included in this study (133 MSF and 533 cementless BHA). One hundred fifty-six hips were divided into nondisplaced FNF (Group A) and 510 into displaced FNF (Group B). We evaluated (1) surgery-related parameters (anesthesia type, time to surgery, operation time, estimated blood loss and volume of postoperative transfusion), (2) the rate of and reasons for reoperation, (3) the rate and type of in-hospital systemic complications and (4) one-year mortality rate after surgery. In Group A, MSF showed shorter operation time (p = 0.004) and lower incidence of in-hospital systemic complications (p = 0.003). In Group B, cementless BHA demonstrated lower reoperation rate than MSF (p < 0.001). In both Group A and B, cementless BHA was associated with higher estimated blood loss than MSF (p < 0.001). Based on findings in our study, MSF might be a more favorable option for nondisplaced FNF, whereas cementless BHA might be a better one for displaced FNF in patients older than fifty. Nevertheless, our nationwide study also showed that numbers of cementless BHAs were being performed for nondisplaced FNF even in teaching hospitals.

## Introduction

The prevalence of hip fractures is rising with the increase in the elderly population^1–4^. Femoral neck fracture (FNF) has been reported to account for approximately half of all hip fractures^5^, and surgery is considered the standard treatment for FNF because of the higher mortality and morbidity associated with conservative treatment^6^. Based on complication rates, the traditional options for surgical treatment have been proposed as multiple screw fixation (MSF) for nondisplaced FNF^7^ and cemented bipolar hemiarthroplasty (BHA) for displaced FNF in elderly patients^8,9^. In spite of higher risk of reoperation for any reason^10^ and periprosthetic fractures^11^, cementless BHA recently gained popularity as a treatment for FNF in elderly patients because of its shorter operation time, reduced blood loss, and lower medical expenditure when compared with cemented BHA^11–14^. However, there have been few studies comparing MSF and cementless BHA for FNF in the elderly population, and it is not clear whether this previous binary strategy for displaced and nondisplaced FNF can be extended to cementless BHA^8,15,16^. Moreover, most studies comparing MSF and BHA for FNF in elderly patients were limited because they lacked a comprehensive approach that evaluated factors related to the surgery itself, reoperation rate, in-hospital systemic complications, and mortality after surgery and included only small numbers of patients^15,17^.

Therefore, the purpose of this study was to compare MSF and cementless BHA as treatments for FNF using nationwide data and to evaluate (1) surgery-related parameters including anesthesia type, time to surgery, operation time, estimated blood loss, and amount of transfusion, (2) the reoperation rate as a local complication, (3) the rate and type of in-hospital systemic complications, and (4) the mortality rate at 1 year after surgery.

## Materials and methods

### Study design and ethical considerations

This multicenter cohort study was performed in accordance with the Code of Ethics of the World Medical Association (Declaration of Helsinki). Approval for the study was obtained from the concerned Institutional Review Board at each study site. Patients were informed that their medical data would be used for scientific studies, and written informed consent was obtained from all patients before the study.

### Participants and data collection

Data of the patients in this study were extracted from the Korean Hip Fracture Registry, an osteoporosis-related hip fracture network incorporating 16 university hospitals in South Korea. The cohort was established in July 2014, and the data were collected prospectively at the central office using the Korea National Institute of Health web-based system (Internet-based Clinical Research and Trial Management system, iCreaT; Cheongju, Korea). Participant assessment and data collection were conducted by a trained interviewer following the same guidelines at each institution. In addition to anteroposterior and lateral X-rays, the collected data included the patients’ sex, age, body mass index (BMI), type and number of comorbidities, ambulatory level before the fracture, general health status at the time of surgery, cause and location of the fracture, type of fracture, time to surgery, anesthesia and surgical details, estimated blood loss, amount of blood transfused, perioperative complications, and annual mortality, among others.

**Figure 1:**
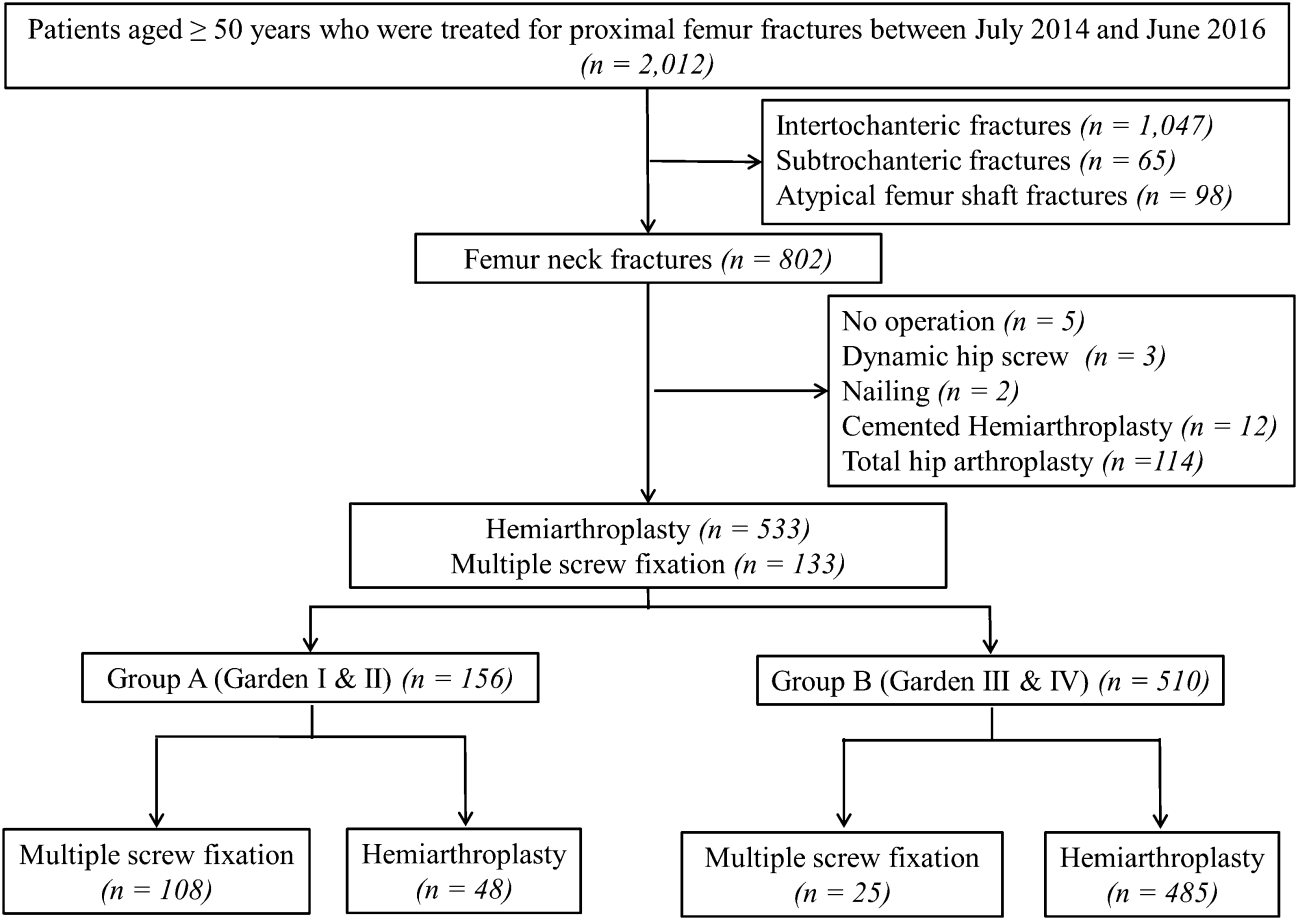
Flow chart to identify multiple screw fixation and cementless hemiarthroplasty for treating femoral neck fractures among Korean Hip Fracture Registry.

From July 2014 to June 2016, a total of 2,012 adults aged ≥ 50 years who had a fragility fracture around the hip joint (excluding pathological fracture) were enrolled in the registry (Fig. 1). All fractures were performed at tertiary referral hospital by hip specialty, who dedicated to operating solely on the hip. The inclusion criteria for this study were (1) patients who sustained FNF due to low-energy trauma^18^ and (2) those who underwent MSF or cementless BHA. The type of surgery to be performed was determined by the individual surgeon’s preference, which was based on the fracture type according to the Garden classification^19^, presence of comminution, patients’ age, activity levels before injury, and number of comorbidities. Exclusion criteria were patients with intertrochanteric fracture (1,047 hips), subtrochanteric fracture (65 hips), and atypical fracture (98 hips). Patients with FNF who underwent total hip arthroplasty (114 hips), cemented BHA (12 hips), compression hip screw (3 hips), or femoral nail (2 hips) or those who did not undergo any operation (5 hips) were also excluded. Thus, the remaining 666 hips (MSF in 133 hips and cementless BHA in 533 hips) were included in this study. There were 465 women and 201 men, and their mean age at the time of index operation was 76.8 ± 9.5 years. The average BMI was 22.0 ± 3.4 kg/m^2^, and the mean duration of follow-up was 29.4 ± 7.7 months (Table 1).

**Table 1:** Comparison of demographic data according to fracture pattern and type of surgery.

Parameters	Group A			*p*-value**	Group B			*p*-value††	*p*-value^§§^
	All *n* = *156*	MSF *n* = *108*	BHA *n* = *48*		All *n* = *510*	MSF *n* = *25*	BHA *n* = *485*		
Sex (male:female), patients	49:107	31:77	18:30	0.182	152:358	7:18	145:340	0.520	0.386
Age, years*	72.9 ± 11.0	69.9 ± 11.2	79.7 ± 6.9	< 0.001	78.0 ± 8.6	62.0 ± 10.7	78.8 ± 7.7	< 0.001	< 0.001
Follow-up duration, months*	29.4 ± 5.7	29.1 ± 5.0	29.7 ± 4.7	0.125	27.8 ± 6.5	27.1 ± 4.7	28.5 ± 5.2	0.135	0.072
BMI, kg/m^2^*	21.8 ± 3.3	21.9 ± 3.4	21.7 ± .3.3	0.804	22.1 ± 3.4	22.4 ± 3.3	22.1 ± 3.4	0.668	0.852
**No. of comorbidities (CCI)**									
0	74 (47.4%)	51 (47.2%)	23 (47.9%)	0.495	242 (47.5%)	12 (48%)	230 (47.4%)	0.582	0.553
1	55 (35.3%)	37 (34.3%)	18 (37.5%)		175 (34.3%)	9 (36%)	166 (34.2%)		
2	21 (13.5%)	15 (13.9%)	6 (12.5%)		64 (12.5%)	3 (12%)	61 (12.6%)		
≧3	6 (3.8%)	5 (4.6%)	1 (2.1%)		29 (5.7%)	1 (4%)	28 (5.7%)		
Osteoporosis, patients (%) (≤ T-score − 2.5)	76 (59.8%)	48 (44.4%)	28 (58.3%)	0.015	284 (55.7%)	10 (40%)	274 (56.5%)	0.043	0.071
**Walking ability, patients (%)**^**§**^									
Outdoor (Koval’s grade I-III)	132 (84.6%)	97 (89.8%)	35 (72.9%)	0.008	404(79.2%)	24(96%)	380 (78.4%)	0.020	0.136
Housebound (Koval’s grade IV-VI)	24 (15.4%)	11 (10.2%)	13 (27.1%)		106(20.8%)	1(4%)	105 (21.6%)		
**ASA score, patients (%)**									
1 and 2	98 (62.8%)	71 (65.7%)	27 (56.3%)	0.116	251(49.2%)	22(88%)	229 (47.2%)	< 0.001	0.003
3 and 4	58 (37.2%)	37(34.3%)	21(43.7%)		259(50.8%)	3(12%)	256(52.8%)		

*Expressed as average ± standard deviation.

^§^Modified from Koval’s grade^15^.

**Significance between MSF and BHA within Group A.

^††^Significance between MSF and BHA within Group B.

^§§^Significance between Group A and B.

MSF, multiple screw fixation; BHA, cementless bipolar hemiarthroplasty; *n*, numbers; BMI, body mass index; ASA, American Society of Anesthesiologists; CCI, Charlson’s Comorbidity Index.

### Evaluation

X-rays were taken before and immediately after surgery and at each outpatient’s visit thereafter and were evaluated by two independent orthopedic surgeons who did not participate in the surgery. At each institution, a trained interviewer assessed the preinjury ambulatory level from the patients and family members and then classified the patients as outdoor or indoor ambulators, modified based on Koval’s grade^20^. A recent study^21^ reported that it is important for ambulators with walking limitations to walk around the community to maintain physical function, and this might be in accordance with our assumption that outdoor ambulators who used walking aids may have better function and lower mortality than others. Therefore, we simplified and categorized the preinjury walking level into outdoor and indoor walkers. The type and number of comorbidities were determined by reviewing the patients’ medical records and by interviewing the patients and family members in each institution. General health status was assessed using the American Society of Anesthesiologists (ASA) score^22^. The type of fracture and surgery were reviewed and reported by the operating surgeons.

Surgery-related parameters included type of anesthesia, time to surgery, operation time, estimated blood loss^23^, and volume of postoperative transfusion, all of which were determined from the operative records. Time to surgery was defined as the elapsed time from injury to index operation, and operation time was calculated from skin incision to closure.

Reoperation rate as a local complication and reasons for reoperation were also evaluated from X-rays and medical records. Despite local complications necessitating reoperation, some patients or family members refused to undergo further surgery because of the patient’s poor general condition or financial problems. The presence and type of systemic complications during hospitalization were also collected from medical records. One-year mortality rate after surgery was investigated using hospital records and/or by interviewing the patient’s family. A systemic search for a death certificate at the National Statistical Office was conducted for patients who were lost to follow-up^24^.

### Patient groups

According to the Garden classification^19^, those with stage I and II were classified into nondisplaced FNFs as Group A (156 hips; 108 MSF, 48 cementless BHA), whereas those with stage III and IV were categorized into displaced FNFs as Group B (510 hips; 25 MSF, 485 cementless BHA). Demographic characteristics, duration of follow-up, number of comorbidities based on the modified Charlson Comorbidity Index^25^, presence of osteoporosis using bone mineral density (T-score ≤ -2.5)^26^, walking ability before injury, and ASA score^22^ were compared between the two groups (Table 1).

### Statistical analysis

We analyzed the data using an independent t-test for continuous variables and chi-square test or Fisher exact test for categorical variables. All database management and statistical analyses were performed using SPSS software version 22.0 (IBM Corp., Armonk, NY, USA). All reported p values were two-sided, and a p-value of < 0.05 was used to determine significance.

## Results

### Demographic characteristics of two groups

Patients with displaced FNF (Group B) were older (p < 0.001) and had a higher risk for anesthesia than those with a nondisplaced FNF (Group A; p = 0.003) (Table 1). As compared with those with MSF, patients undergoing cementless BHA were older (p < 0.001) and had a higher prevalence of osteoporosis and worse walking ability before injury in each group (p < 0.05). Sex, follow-up duration, BMI, and number of comorbidities were not different regardless of the type of fracture or surgery (p > 0.05).

### Surgery-related parameters

Time to surgery was longer in patients with cementless BHA than in those with MSF in Group A (5.4 and 3.1 days, p = 0.003) and Group B (5.3 and 2.7 days, p < 0.001; Table 2). Operation time was longer for cementless BHA than MSF in Group A (69.1 and 53.4 min, p = 0.004) but was not different in Group B (p = 0.934). Estimated blood loss was greater for cementless BHA than MSF in Group A (241.5 and 107.3 mL, respectively) and Group B (275.3 and 87.2 mL, respectively; p < 0.001). The type of anesthesia and postoperative transfusion volume were not different between cementless BHA and MSF in both groups.

**Table 2:** Comparison of surgery-related parameters between patients with MSF and cementless BHA according to fracture type.

Parameters	Group A		*p*-value	Group B		*p*-value
	MSF *n* = *108*	BHA *n* = *48*		MSF *n* = *25*	BHA *n* = *485*	
**Type of anesthesia, patients (%)**			0.497			0831
Regional	65 (60%)	12 (25%)		16 (64%)	320 (66%)	
General	43 (40%)	36 (75%)		9 (36%)	165 (34%)	
Time to surgery, days*	3.1 ± 3.4	5.4 ± 4.5	0.003	2.7 ± 3.3	5.3 ± 5.5	< 0.001
Operation time, minutes*	53.4 ± 37.1	69.1 ± 26.9	0.004	69.1 ± 82.5	69.6 ± 25.6	0.934
Estimated blood loss, mL*	107.3 ± 180.8	241.5 ± 178.6	< 0.001	87.2 ± 141.0	275.3 ± 233.2	< 0.001
Volume of transfusion, mL*	28.5 ± 144.2	73.1 ± 173.1	0.096	12.0 ± 44.0	80.7 ± 203.8	0.093

*Expressed as average ± standard deviation.

^†^Surgical approach statistically compared between Group A and B.

MSF, multiple screw fixation; BHA, cementless bipolar hemiarthroplasty; *n*, numbers.

### Reoperation rate as a local complication

The reoperation rate was not different in Group A (2.8% and 2.1%, respectively, p = 0.639), but was significantly higher in MSF than cementless BHA in Group B (24.0% and 2.9%, p < 0.001; Table 3). The main reasons for reoperation in each group were nonunion and osteonecrosis of the femoral head (ONFH) in MSF and postoperative periprosthetic fracture in cementless BHA. Seven patients (78%) with complicated MSF underwent arthroplasty, whereas 6 patients (40%) with compromised cementless BHA received revision or conversion arthroplasty.

**Table 3:** Main causes of reoperation in patients with MSF and cementless BHA according to fracture type.

Cause of reoperation	Group A		*p*-value	Group B		*p*-value
	MSF *n* = *108*	BHA *n* = *48*		MSF *n* = *25*	BHA *n* = *485*	
Total, hips (%)	3 (2.8%)	1 (2.1%)	0.639	6 (24%)	14 (2.9%)	< 0.001
Periprosthetic fracture, hips (%)	0	1 (2.1%)	0.132	0	11 (2.3%)	0.447
Nonunion, hips (%)	2 (1.9%)	0	0.343	3 (12%)	0	< 0.001
Dislocation, hips (%)	0	0	NA	0	2 (0.4%)	0.748
Infection, hips (%)	0	0	NA	0	1 (0.2%)	0.820
Osteonecrosis, hips (%)	1 (0.9%)	0	0.504	3 (12%)	0	< 0.001

MSF, multiple screw fixation; BHA, cementless bipolar hemiarthroplasty; *n*, numbers; NA, not applicable.

### In-hospital systemic complications

The incidence of systemic complications during hospitalization was higher in patients with cementless BHA than in those with MSF in Group A (13.0% and 31.2%, respectively, p = 0.003; Table 4). Regardless of the type of fracture or operation, delirium was the most common in-hospital systemic complication after surgery (12.6%, 84/666 hips) and occurred more frequently in patients undergoing cementless BHA (p = 0.015 in Group A and p = 0.048 in Group B). Except for acute renal failure, other complications did not differ between surgeries in each group (Table 4).

**Table 4:** Comparison of in-hospital systemic complications between patients with MSF and cementless BHA according to fracture type.

Complications	Group A		*p*-value	Group B		*p*-value
	MSF *n* = *108*	BHA *n* = *48*		MSF *n* = *25*	BHA *n* = *485*	
Total*,* hips *(%)*	14 (13.0%)	15 (31.2%)	0.003	2 (8.0%)	111 (22.9%)	0.081
Delirium, hips (%)	8 (7.4%)	10 (20.8%)	0.015	0	66 (13.6%)	0.048
Pneumonia, hips (%)	1 (0.9%)	3 (6.3%)	0.052	1 (4%)	22 (4.5%)	0.900
Urinary tract infection, hips (%)	0	1 (2.1%)	0.132	0	10 (2.1%)	0.468
Venous thromboembolism	3 (2.7%)	0	0.244	0	8 (1.6%)	0.517
Voiding difficulty, hips (%)	1 (0.9%)	0	0.504	0	5 (1.0%)	0.610
Acute renal failure, hips (%)	1 (0.9%)	1 (2.1%)	0.553	1 (4%)	0	< 0.001

MSF, multiple screw fixation; BHA, cementless bipolar hemiarthroplasty; *n*, numbers.

### One-year mortality rate

The mortality rate at 1 year after surgery was not different based on fracture pattern or type of surgery (p > 0.05); Group A demonstrated a 1-year mortality rate of 8.3% after MSF (9/108 hips) and 8.3% after cementless BHA (4/48 hips, p = 0.634), whereas Group B had a mortality rate at 1 year of 4.0% after MSF (1/25 hips) and 8.2% after cementless BHA (40/485 hips, p = 0.385).

## Discussion

In spite of recent popularity of cementless BHA as a treatment for FNF, there have been no comprehensive studies comparing it with MSF in the elderly population. Our nationwide study showed that MSF had shorter operation time, less blood loss and fewer in-hospital systemic complications in nondisplaced FNF whereas cementless BHA had a lower reoperation rate in displaced FNF. The annual mortality rate did not differ regardless of surgery type. It was remarkable in our study that 30.7% (48/156) of patients with nondisplaced FNF underwent cementless BHA on the national level.

Despite a shorter time to surgery to decrease further displacement and a reduction in subsequent complications when compared with BHA after preoperative optimization^27^, the time to MSF was 3.1 days in nondisplaced and 2.7 days in displaced FNF, which was longer than the 24 to 48 h recommended by previous studies^28,29^(Table 2). This might originate from the characteristics of the participating institutions (university hospitals) as a referral center, resulting in more time taken for referral and management of severe comorbidities. This delay in part might affect the incidence of postoperative complications after MSF.

The operation time was shorter in MSF in nondisplaced FNF (p = 0.004) but did not significantly differ between groups in displaced FNF (p = 0.934). Nevertheless, the estimated blood loss was lower in those with MSF in both the groups (p < 0.001). Previous studies showed a shorter operation time in cementless BHA when compared with cemented BHA^11,13,30,31^. In displaced FNF, the reduction before MSF, is mandatory and take time, but usually performed using non- to minimally-invasive methods. Thus, we speculated that these led to similar operation time but less estimated blood loss in MSF for displaced FNF when compared to those in cementless BHA.

The reoperation rate in nondisplaced FNF was not different in both MSF and cementless BHA groups (2.8% vs 2.1%, p = 0.639). However, despite their younger age and better bone quality, patients undergoing MSF had a higher reoperation rate than those with cementless BHA in displaced FNF (24.0% and 2.9%, p < 0.001; Tables 1 and 3). The main reasons for reoperation were nonunion and ONFH after MSF and postoperative periprosthetic fracture after cementless BHA regardless of fracture type. These findings in our study are similar to those of previous reports after MSF or cemented BHA^32–36^ and thus, cementless BHA might be a more favorable option for displaced FNF over MSF.

Patients with cementless BHA had or tended to have a higher incidence of in-hospital systemic complications than those with MSF (p = 0.003 in nondisplaced and p = 0.081 in displaced FNF; Table 4); delirium was the most common in-hospital systemic complication and occurred more frequently in patients with cementless BHA than in those with MSF (p = 0.015 in nondisplaced and p = 0.048 in displaced FNF). In nondisplaced FNF, the significant difference in the rate of delirium between MSF and cementless BHA might be related to older age and worse preoperative walking ability, which reflects physical function in patients with cementless BHA and, in part, might originate from more blood loss during cementless BHA. Also, time to surgery was longer in patients with cementless BHA than in those with MSF and this time delay of 2 days might partly account for the increased rate of delirium in cementless BHA group^37^. In displaced FNF, however, because MSF and cementless BHA have similar operation time, this significance of the difference seems to become less. Indeed, delirium in elderly patients with FNF should be monitored more carefully. Mortality rate at 1 year was not different after MSF and cementless BHA in each group (p > 0.05) and compatible with the findings of previous studies^38,39^.

Although our study showed that MSF had shorter operation time, less blood loss and fewer in-hospital systemic complications in nondisplaced FNF, our nationwide study also demonstrated that many surgeons even in teaching hospitals were performing cementless BHA for nondisplaced FNF (31% in Table 1). A recent randomized controlled study demonstrated that BHA for nondisplaced FNF improved mobility and fewer reoperation (5% vs 20%)^15^ and some studies reported that cementless BHA showed shorter operation time and reduced blood loss when compared with cemented BHA^11–14^. We speculated that these might mislead some surgeons to favor cementless BHA over MSF in nondisplaced FNF and thus, our study may be valuable in that this provides some implications to the current clinical practice.

Of course, our study has several limitations. First, although the data in this nationwide study was gathered in a prospective manner, this was a retrospective study. Second, the incidence of ONFH after MSF might be underestimated; because only simple radiographs were collected for radiographic evaluation in the Korean Hip Fracture Registry, we might not identify ONFH with early stage after MSF. Moreover, relative short-term duration of follow-up might be not enough to identify ONFH. However, the average time to take for diagnosis of ONFH after FNF has been reported as about 1 year^16^ and thus, most of ONFH after MSF might be identified during the study period. Third, in spite of local complications that might require reoperation, some patients or family members might refuse to undergo further surgery because of poor general condition or financial problems. Thus, the reoperation rate may have been underestimated in our study. Nevertheless, we believe that our study is valuable in that it was performed based on national level comparing the outcome after MSF or cementless BHA which showed shorter operation time and gained recent popularity as a treatment for FNF.

In conclusion, MSF might be a more favorable option for nondisplaced FNF because of reduced operation time, blood loss and in-hospital systemic complications, whereas cementless BHA might be a better one for displaced FNF in terms of its lower reoperation rate in patients older than fifty. Nevertheless, our nationwide study also showed that numbers of cementless BHAs were being performed for nondisplaced FNF even in teaching hospitals.

### Ethical approval

This article does not contain any studies with human participants or animals performed by any of the authors. The design and protocol of this study were approved by the Institutional Review Board (IRB) approval (EMCS 2020–03-010).

### Informed consent

Informed consent was obtained from all individual participants included in the study.

## Data Availability

The datasets generated during and/or analyzed during the current study are available by request.
